# Production of Tetramethylpyrazine from Cane Molasses by *Bacillus* sp. TTMP20

**DOI:** 10.3390/molecules28062640

**Published:** 2023-03-14

**Authors:** Yujia Li, Lirong Luo, Xiaoxiao Ding, Xiumin Zhang, Shanling Gan, Changhua Shang

**Affiliations:** Key Laboratory of Ecology of Rare and Endangered Species and Environmental Protection, Guangxi Normal University, Ministry of Education, Guangxi Key Laboratory of Landscape Resources Conservation and Sustainable Utilization in Lijiang River Basin, Guangxi Normal University, Guilin 541006, China; liyujia@stu.gxnu.edu.cn (Y.L.); gxnu202011002019@stu.gxnu.edu.cn (L.L.); dxx107@stu.gxnu.edu.cn (X.D.); zxm1413655310@163.com (X.Z.); ganshanling1999@163.com (S.G.)

**Keywords:** *Bacillus* sp. TTMP20, tetramethylpyrazine, cane molasses, pretreatment

## Abstract

2,3,5,6-Tetramethylpyrazine (TTMP) is an active ingredient of *Ligusticum wallichii* Franch. It can be used in medicine and food fields. In this study, *Bacillus* sp. TTMP20 was applied to produce TTMP using cane molasses as a carbon source. After pretreatment with phosphoric acid, 170 mL/L treated molasses, combined with 10 g/L yeast powder, 30 g/L tryptone and 30 g/L (NH_4_)_2_HPO_4_ were used for fermentation. After 36 h, TTMP output reached the highest value of 208.8 mg/L. The yield of TTMP using phosphoric acid-treated molasses as carbon source was 145.59% higher than control. Under the sulfuric acid treatment process of molasses (150 g), the maximum yield of TTMP was 895.13 mg/L, which was 183.18% higher than that of untreated molasses (316.1 mg/L). This study demonstrated that molasses is a high-quality and inexpensive carbon source for the manufacture of TTMP, laying the groundwork for the future industrial production of TTMP.

## 1. Introduction

2,3,5,6-Tetramethylpyrazine (TTMP) is an active substance of *Ligusticum wallichii* Franch (named as Chuan Xiong by Chinese), one of the traditional Chinese medicinal herbs [1]. Chuan Xiong was used to cure many cardiovascular diseases 1500 years ago. The effect of TTMP on cardiovascular diseases such as atherosclerosis and hypertension has been extensively studied [2].

In addition, TTMP has a pleasant tonality of nutty and toasty, so TTMP has been used as a flavoring additive in food industry. It is also found in a variety of fermented foods including Chinese liquors [3]. The previous research on liquor has found that TTMP is not only the main contributor to Jiang flavor of Chinese liquors but also an important active pharmaceutical ingredient [4]. Kosuge has found TTMP in Japanese fermented soybean, and TTMP derived from microorganisms [5].

Many studies have proven that TTMP could be synthesized in bacteria, which provided the practical basis for biosynthesis [6,7,8]. The demand for green products has been rising with the improvement of living standards and environmental awareness. Biotechnological production is a green synthesis pathway for TTMP production [9]. Compared with the traditional methods such as the extraction from herbs and chemical synthesis, biotechnological production has the following advantages: simple operation, cyclic utilization, mild reaction conditions and an environment-friendly way.

Molasses is a kind of by-product in the sugar-making industry, that has high level of sugar and vitamins [10]. Molasses is a promising carbon source with a significant content of fermentable sugars [11]. In addition, as a cheap and environment-friendly substrate, molasses is widely used to produce many compounds such as succinic acid, glutamic acid, ethanol and 2,3-butanediol [10,12,13,14,15,16].

Guangxi Autonomous Region is the main sugarcane production base in China. The sugar industry is the main contributor to the local financial revenue in Guangxi [17]. In China, most of molasses was simply abandoned, which caused heavy environmental pollution. Molasses contains a substantial amount of nutriment, which can be utilized in fermentation. Proper treatment of molasses can not only reduce the cost of waste disposal, but also gain additional benefits [18].

## 2. Results and Discussion

### 2.1. Isolation of Bacterial Strain

The strain with high content of TTMP used in this study was isolated from high-temperature Jiang flavor Daqu of Danquan distillery in Nandan county, Hechi city, Guangxi based on the production ability of acetoin (the precursor of TTMP biosynthesis) and the final yield of TTMP. Based on the study of the 16S rDNA sequence, the strain was identified as a Bacillus. Hence, the strain was designated as Bacillus species TTMP20.

### 2.2. Composition of Cane Molasses

Based on the test, cane molasses contained the following components (*w*/*w*): total sugar of 37.77%, water of 25.24%, reduced sugar of 8.55%, crude protein of 13.25%, ash of 8.4%, and it had a pH value of 4.84. Table 1 described the chemical composition of cane molasses. The former studies of Li et al. and Wu et al. reported the chemical composition of Guangxi cane molasses, which contained (*w*/*v*) sucrose of 45%, converted sugars of 10% (glucose of 5% and fructose of 5%), crude protein of 1.5%, ash of 5.1%, salt of 2.6%, and water of 25% [19,20]. Compared with other cane molasses, there was a certain gap in crude protein, which was attributed to the different technological processes or materials. In summary, many factors might influence the nutrient content, color, and viscosity of molasses, including the varieties of sugarcane, the environment of production area of raw material, process technology and conditions of the factory. Sugar was the main component of cane molasses.

### 2.3. Change of Molasses Composition after Treatment

Molasses, as a natural and cheap material, is widely used in fermentation. Inorganic salts, organic acids, polyphenols and other active substances in molasses can promote microbial fermentation to a certain extent [21]. However, molasses is a by-product of sugar production, and the pigments, ashes and metal ions will affect the growth of microorganisms in molasses.

To effectively improve cell density and the content of fermentation products, pretreatment of raw molasses is necessary for fermentation. Effective pretreatment can reduce the hazardous substances in molasses and maximize the utilization rate of sugar. Several treatments were compared, which were easy to operate and can selectively remove the components affecting fermentation in molasses.

In both the ST and PT methods, the steps are the same, except that the acid adjustment pH is different. The molasses is heated, which causes the insoluble colloid, ash and solid impurities to precipitate. Then polysaccharide is hydrolyzed into monosaccharide which is easily exploited by microorganisms, and the colloid and ash are further precipitated. The pH is adjusted to near neutral with calcium hydroxide. The pigments and biological macromolecules were removed by adding active carbon, and the color was adsorbed to clarify the molasses [22]. In many studies of molasses fermentation, sulfuric acid was often used to pretreat molasses. Jiang’s research [23] on butyric acid fermentation by *Clostridium tyrobutyricum* found that butyric acid concentration, yield and sugar utilization were greatly improved by using sulfuric acid to pretreat molasses. It has been proven that sulfuric acid-heat treatment is effective in enhancing bacterial cellulose production. With sulfuric acid-heat treatment, the maximum bacterial cellulose concentration increased to 76% more than that obtained using untreated molasses [24]. Lazaridou et al. [25] used sulfuric acid and activated carbon to remove the fermentation inhibitor in molasses. Under the optimal conditions, the utilization rate of sugar reached 92%, and the maximum concentration of pullulan reached 24 g/L.

The composition of treated molasses is shown in Table 2. Total sugar content of the PT method was the lowest among all treatment methods, which was 41.18% lower than that of the diluted molasses. It can be inferred that phosphoric acid and calcium hydroxide generate a large amount of calcium phosphate, and calcium phosphate can effectively remove colloid, ash and some pigments in molasses, but also lead to a large amount of sugar loss. The content of colloid in molasses decreased by 0.42% and the content of ash decreased by 3.63% when the molasses was treated with phosphoric acid and polyacrylamide [26].

*Bacillus* sp. TTMP20 was inoculated into medium containing PT-treated molasses. Compared with other treatments, the growth of bacteria had no obvious advantage (Table 2). The ST method was a relatively advantageous treatment method, that did not cause a large amount of sugar loss. After treatment, total sugar only decreased by 5.13%, and the content of reduced sugar effectively increased by 30.7% (Table 2). Under ST method, there was the highest cell density of *Bacillus* sp. TTMP20 in all treatment methods.

Molasses with different treatment methods (170 mL/L) was added into culture medium. Diluted molasses (DM) represents untreated molasses. ST, PT, PFT, COT and CT represent sulfuric acid treatment process, phosphoric acid treatment process, potassium ferrocyanide treatment process, calcium phosphate tribasic treatment process, combinational treatment process, respectively. Data were obtained from triplicate tests and presented as mean ± SD.

Potassium ferrocyanide can efficiently take off heavy metal ions in molasses. It can form complex precipitation with protein and other colloidal substances in molasses, and a large number of impurities can also be removed during precipitation [27]. Under PFT method, all sugar content was reduced, and there was no obvious advantage compared with other treatment methods (Table 2). In addition, potassium ferrocyanide residue in molasses might inhibit fermentation [28,29].

Calcium phosphate tribasic is also often used to treat molasses. For example, He et al. and Kundu et al. found that calcium phosphate tribasic could reduce the level of inorganic and organic inhibitors in molasses [30,31]. Küçükaşik et al. studied different pretreatment methods of molasses on cell growth of *Halomonas* sp. AAD6 and levan production [32]. They found that calcium phosphate tribasic treatment could selectively and efficiently remove iron and zinc in molasses or other similar mixtures. Roukas found that beet molasses treated with calcium phosphate tribasic treatment not only improved the utilization rate of sugar, but also increased the yield and concentration of polysaccharide [33]. In our study, COT method could greatly increase the content of total sugar, perhaps because the added calcium phosphate tribasic absorbed part of the water and caused the loss of water when removing the precipitation. The COT method also increased the content of reducing sugar. The molasses treated with calcium phosphate tribasic had a certain potential in cultivating microorganisms, and OD value of *Bacillus* sp. TTMP20 reached 1.92 after 36 h (Table 2).

The CT method was the most complex among all treatment methods and effectively improved the content of reduced sugar. In the CT method, reduced sugar content increased by 83.4%, sucrose content decreased by 45.22 g/L to 14.26 g/L, and glucose content increased by 32.8 g/L to 45.61 g/L (Table 2). The results showed that this treatment method could effectively promote the decomposition of polysaccharides to obtain more reducing sugar. However, the growth of Bacillus sp. TTMP20 was the worst under the CT method, which could be attributed to the complexity of this treatment method. The added substances were not completely removed from the molasses, which inhibited the growth of *Bacillus* sp. TTMP20 (Table 2).

### 2.4. Growth of Strain Bacillus *sp.* TTMP20

**Figure 1:**
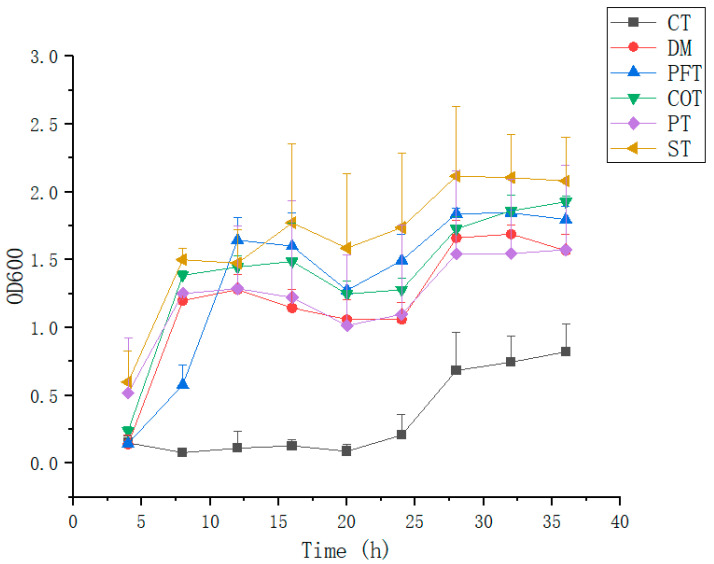
Growth curve of strain *Bacillus* sp. TTMP20 using molasses with different treatment methods as carbon source. Molasses with different treatment methods (170 mL/L) was added into culture medium. The growth contained two stages (47 °C from 0 h to 24 h, 60 °C from 24 h to 36 h). DM corresponds to untreated molasses. ST, PT, PFT, COT and CT represent sulfuric acid treatment process, phosphoric acid treatment process, potassium ferrocyanide treatment process, calcium phosphate tribasic treatment process, combinational treatment process, respectively. Data were obtained from triplicate tests and shown as mean ± SD.

The bacterium *Bacillus* sp. TTMP20 used in this study was isolated from high-temperature Jiang flavor Daqu. The complex flavor of Danquan Baijiu benefited from high-temperature Daqu which derived from a unique brewing technology [34]. During the production of high-temperature Daqu, the highest Daqu temperature can reach above 60 °C, which makes the high-temperature resistant bacteria the dominant microbes in high-temperature Daqu [35]. As shown in Figure 1, only 8 h after inoculation *Bacillus* sp. TTMP20 was in the vigorous growth stage. There was no obvious change in biomass from 8 h to 24 h. After 24 h of fermentation, raise fermentation temperature to 60 °C. We found that biomass increased after an increase in temperature, and achieved the maximum after 36 h of fermentation. Perhaps the high temperature activated the spore. In the study of using molasses to produce succinic acid, it was found that sulfuric acid could remove heavy metals and suspended impurities in molasses, and culture medium with molasses treated with sulfuric acid was more favorable for cell growth [36].In this study, cell growth in molasses pretreated with sulfuric acid(ST) was the fastest in the early stage (0–4 h), and the biomass was the largest at the end of fermentation. The biomass of the combined treatment(CT) was the least, which might be attributed to the complexity of CT method. It not only fails to effectively remove the impurities that inhibit cell growth in molasses, but also retains the substances during the treatment in molasses and inhibits cell proliferation.

### 2.5. Analysis of Sugar Metabolism

**Figure 2:**
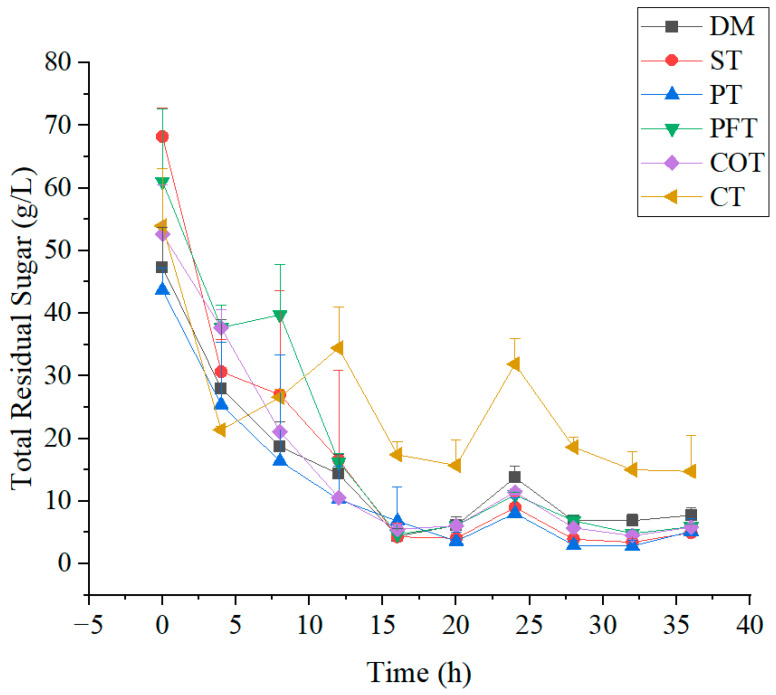
Changes in total residual sugar in culture medium containing molasses with different treatment methods. Molasses with different treatment methods (170 mL/L) was added into culture medium. DM represents untreated molasses. ST, PT, PFT, COT and CT represent sulfuric acid treatment process, phosphoric acid treatment process, potassium ferrocyanide treatment process, calcium phosphate tribasic treatment process, combinational treatment process, respectively. Data were obtained from triplicate tests and shown as mean ± SD.

**Figure 3:**
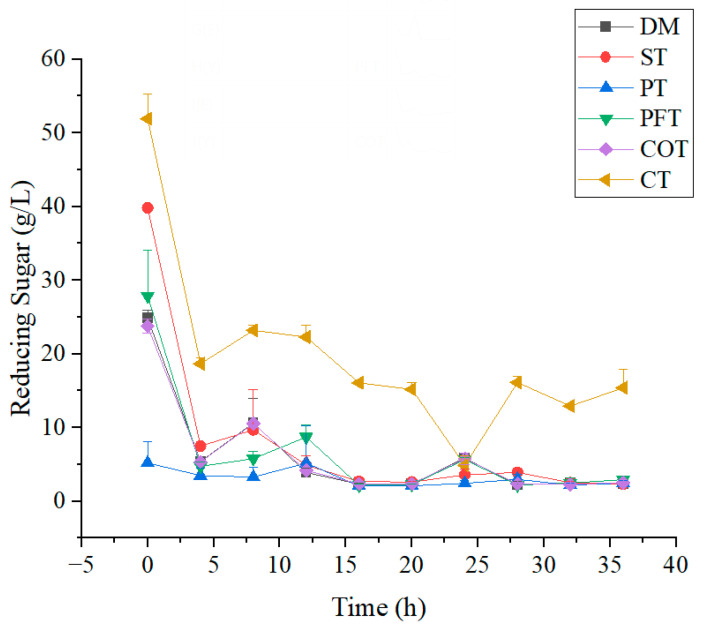
Changes in reducing sugar in culture medium containing molasses with different treatment methods. Molasses with different treatment methods (170 mL/L) was added into culture medium. DM represents untreated molasses. ST, PT, PFT, COT and CT represent sulfuric acid treatment process, phosphoric acid treatment process, potassium ferrocyanide treatment process, calcium phosphate tribasic treatment process, combinational treatment process, respectively. Data were obtained from triplicate tests and shown as mean ± SD.

During the fermentation of TTMP, sugar consumption was investigated (Figure 2and Figure 3). In the initial period of fermentation (0–4 h), strain *Bacillus* sp. TTMP20 entered the acceleration period before the logarithmic phase, and some cells have adapted to the new environment and started to reproduce. Strain *Bacillus* sp. TTMP20 preferentially used reducing sugar, and the content of reducing sugar decreased rapidly. In fermentation anaphase, the change of reducing sugar tends to be flat, perhaps reducing sugar has been exhausted. Glucose is the direct carbon source for bacteria to produce TTMP, and acetoin can be produced through glycolysis, pyruvate conversion and other processes [37].

In the first 8 h, the amount of *Bacillus* sp. TTMP20 increased rapidly, and sugar was also rapidly consumed. At this stage, the consumption rate of sugar has reached 3.5 g/L/h. From 0 to 16 h, total sugar content continued to decline and the number of *Bacillus* sp. TTMP20 continued to increase. At this stage, the growth of *Bacillus* sp. TTMP20 was relatively vigorous, and sugars were mainly used for microbial proliferation and anabolism. Pretreatment could effectively improve the utilization of sugars. The utilization rate of untreated molasses was only 83.5%, and the highest and lowest utilization rates of molasses treated with ST method and the combined treatment were 92.6% and 72.6%, respectively. The utilization rate of other pretreated molasses exceeded 88%, which indicated that the appropriate pretreatment methods could effectively improve the utilization rate of sugars derived from molasses.

### 2.6. Utilization of Acetoin

Acetoin (3-hydroxy-2-butanone) is a volatile compound. Its production methods include chemical synthesis and biological fermentation. Acetoin had extensive and in-depth applications in the fields of food industry, cosmetic manufacturing, medicine and the chemical industry [38]. Acetoin is a precursor of several compounds such as TTMP, 2,3-butanediol, liquid hydrocarbon fuels and heterocyclic compounds [39]. Therefore, the utilization of acetoin can indirectly reflect the production of TTMP. The earlier research found that in an environment containing fermentable carbon sources, many microorganisms could synthesize acetoin during glycolysis to avoid acidosis [40]. In the glycolytic pathway of microorganisms, two pyruvate molecules are condensed into one instable α-acetolactate (AL) and carbon dioxide by α-acetolactate synthase (ALS). Acetoin derives from the decarboxylation of AL in the presence of α-acetolactate decarboxylase (ALDC) [40]. Acetoin could be secreted into the growth medium as a carbon source and subsequently reused in the fixed stage when other carbon sources were exhausted [41]. Bacteria can also use exogenous acetoin to synthesize TTMP. Adding an exogenous precursor to culture medium is also a common method to increase the production of TTMP [6]. Metabolic engineering can strengthen the expression of acetoin synthesis related genes, improve the activity of key enzymes, and promote more carbon flux to flow into acetoin synthesis to improve the yield of acetoin, which is also a direction to improve the yield of TTMP at present [42]. Meng et al. constructed a strain over expressing *ALDC* gene [43]. The recombinant strain and the addition of acetaldehyde in fermentation successfully increased the yield of acetoin, and the yield of TTMP also increased by 15.47%.

**Figure 4:**
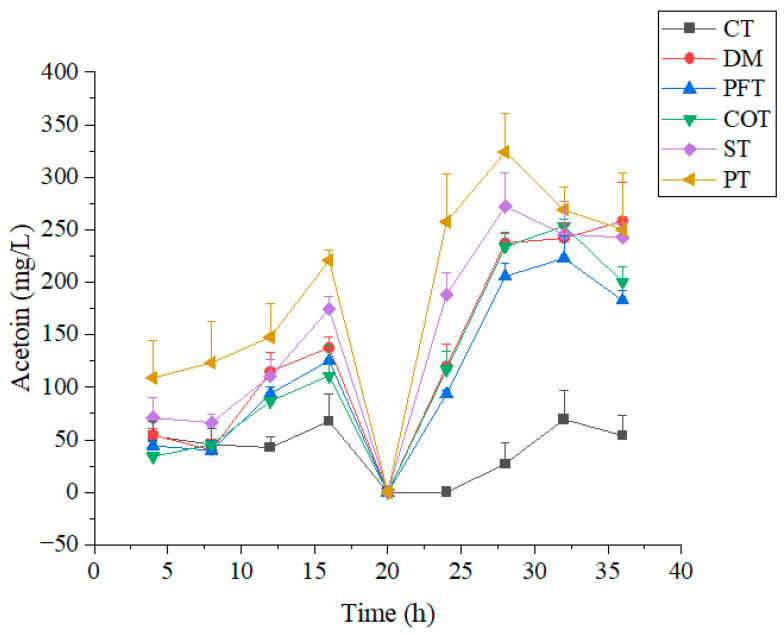
Changes in acetoin in culture medium containing molasses with different treatment methods. Molasses with different treatment methods (170 mL/L) was added into culture medium. DM represents untreated molasses. ST, PT, PFT, COT and CT represent sulfuric acid treatment process, phosphoric acid treatment process, potassium ferrocyanide treatment process, calcium phosphate tribasic treatment process, combinational treatment process, respectively. Data were obtained from triplicate tests and shown as mean ± SD.

From 0 h to 16 h in fermentation (Figure 4), the concentration of acetoin increased with the extension of fermentation time. From 16 h to 20 h, the concentration of acetoin decreased significantly, perhaps acetoin was used as a carbon source when the content of other carbon sources was low in culture medium. From 20 h to 28 h, the content of acetoin continuously increased, reached the highest value at 28 h, and gradually decreased from 28 h to 36 h. At this stage, acetoin was converted to TTMP under a high temperature, which was consistent with the former study [44]. At the end of fermentation, the content of acetoin in fermentation broth was still high, indicating that acetoin in fermentation broth was not completely converted into TTMP, and the conversion rate was low. The maximum residue of acetoin in fermentation broth (control, DM method) was 258 mg/L, and the residue of acetoin in other treatment methods except combined treatment (CT) method also exceeded 150 mg/L. Li et al. constructed cell factories to ferment acetoin, then put fermentation supernatant and diammonium hydrogen phosphate into a high-pressure micro-reactor to synthesize TTMP. Under the optimal conditions, the conversion rate of acetoin reached 85.3% [45]. In the study of solid state fermentation of TTMP from wheat bran, the first step was to add glucose to accumulate acetoin, and the second step was to add diammonium hydrogen phosphate when the concentration of acetoin was at its maximum. Through this strategy, the yield of TTMP increased by 6.8 folds [46].

### 2.7. Production of Tetramethylpyrazine by Fermentation of Molasses with Different Treatments

At present, scientists have found that many microorganisms have the ability to synthesize TTMP, including *Bacillus*, *Corynebacterium glutamicum* and *Saccharomyces cerevisiae* [42,47,48]. *Bacillus* is a kind of microorganism with an obvious advantage in yield, that has been well studied. The synthesis pathway of TTMP in microorganisms has been widely studied, but its synthesis mechanism is still controversial. There are two different views on the mechanism of the conversion of acetoin and ammonia to TTMP. One view suggests that this process is an enzymatic process. However, another view suggests that this is a spontaneous thermodynamic reaction. The second view had sufficient evidence and was supported by most scholars [49,50]. Temperature could affect the conversion of acetoin to TTMP, and high temperature could accelerate this reaction [51]. Therefore, in fermentation experiment, fermentation temperature in the early stage of this study was 47 °C, and after 24 h of fermentation, fermentation temperature was increased to 60 °C to promote the synthesis of TTMP. Moreover, the increasing temperature can inhibit the effect of the enzyme and prevent the decomposition of acetoin by the enzyme [3]. As a by-product of food industry with high sugar content, molasses is a good carbon source for microorganisms. Xiao et al. produced acetoin using molasses as a co-substrate, which showed that molasses was a potential raw material for TTMP fermentation [52].

**Figure 5:**
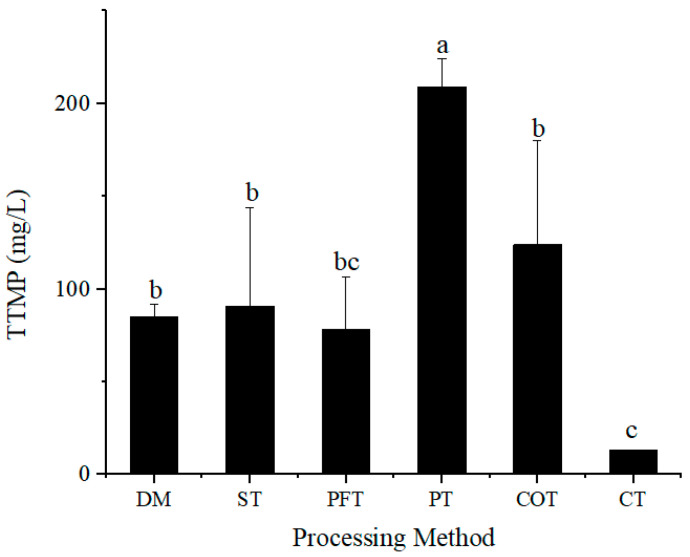
Production of tetramethylpyrazine using molasses with different treatment methods as carbon source. Molasses with different treatment methods (170 mL/L) was added into culture medium. The bar “DM” corresponds to untreated molasses. ST, PT, PFT, COT and CT represent sulfuric acid treatment process, phosphoric acid treatment process, potassium ferrocyanide treatment process, calcium phosphate tribasic treatment process, combinational treatment process, respectively. Data were obtained from triplicate tests and shown as mean ± SD. Bars with a same superscripted letter (a, b or c) mean that there is no statistical difference.

The 170 mL molasses treated by different methods was used to prepare culture medium, and the content of TTMP in fermentation broth was determined after fermentation. The fermentation results are shown in Figure 5. The substances in molasses treated with different treatment methods are different, which resulted in different cell growth efficiency and synthesis efficiency of TTMP. Comparing the yield of TTMP, it was found that not all treatment methods could effectively improve the yield of TTMP. The highest yield of TTMP by fermentation was 208.8 mg/L under PT method and the lowest yield was 13.15 mg/L under CT method. The yield of TTMP using untreated molasses was 85.02 mg/L. The yield of TTMP by phosphoric acid-treated molasses was 145.59% higher than that by untreated molasses, which showed that the effective pretreatment of raw material could greatly increase the output of product. Owing to the inhibition of many additional substances in treatment method, the growth and TTMP yield of strain *Bacillus* sp. TTMP20 under CT method was the least, which indicated that the substances left in molasses not only affected the growth of microorganism but also affected the metabolism of microorganism. There was no statistically significant difference in TTMP yield among ST, PFT, COT and untreated molasses (control, DM).

In this paper, different pretreatment methods were selected to treat molasses. The molasses with the same initial amount had different quality and volume after treatment, which was caused by the complexity of treatment methods and the different substances added in treatment process. In the experiment (Figure 5), the yield of TTMP using the same volume of treated molasses was verified. Next, we explored how much TTMP was produced from molasses with the fixed amount.

**Figure 6:**
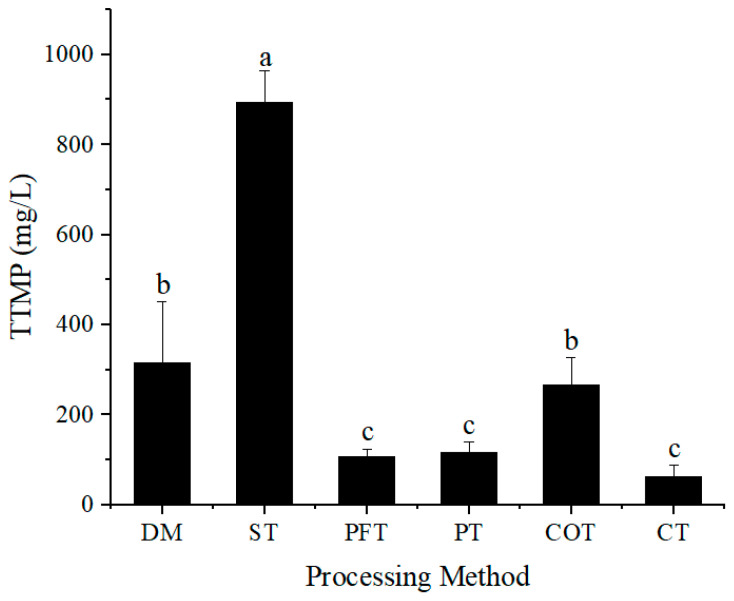
TTMP production from 150 g/L molasses with different treatment methods. DM represents untreated molasses. ST, PT, PFT, COT and CT represent sulfuric acid treatment process, phosphoric acid treatment process, potassium ferrocyanide treatment process, calcium phosphate tribasic treatment process, combinational treatment process, respectively. Data were obtained from triplicate tests and shown as mean ± SD. Bars with a same superscripted letter (a, b or c) mean that there is no statistical difference.

Fix the amount of molasses. First, 150 g molasses were weighed and treated with different methods. All product after treatment was used to prepare culture medium, and the volume of culture medium was fixed to 1 L to explore the TTMP yield of 150 g molasses. During TTMP fermentation, the treatment of molasses could significantly affect the yield of TTMP. After fermentation, the maximum yield of TTMP reached 895.13 mg/L (Figure 6). The highest production of TTMP was obtained under ST treatment of molasses. TTMP production of control group (DM) was 316.1 mg/L, and the yield of other treatments was lower than that of the control. Compared with the test in Figure 5, the added amount of molasses in culture medium was adjusted, resulting in the great changes in TTMP yield. Using molasses treated by ST method, the content of TTMP increased greatly. Using the untreated molasses, the content of TTMP also increased significantly. However, using molasses treated by PFT and COT methods, the contents of TTMP did not increase significantly. Using molasses treated by PT method, the content of TTMP decreased to 116 mg/L. PT treatment had the advantage of fermentation when molasses concentration was low, but when the amount of molasses increased, the production of TTMP in PT treatment decreased. The results in Figure 6 indicated that PT treatment was not suitable for the fermentation of large quantities of molasses.

The researchers have become increasingly interested in an inexpensive substrate for TTMP production in recent years. At present, most of the researches on microbial production of TTMP focus on improving the production of TTMP, but the development of cheap and reliable raw materials for fermentation is also a key point to achieving large-scale industrial production.

Wen et al. obtained 6.93 mg/g DW (dried weight) of TTMP by fermenting adlay (*Coix lacryma-jobi*) [53]. The production capacity of tapioca flour and cottonseed has also been verified, but the process undergoes the complex and long-term pretreatment [45]. In this paper, the ability of molasses to produce TTMP was proved, and the effects of treatment methods of molasses on TTMP production were explored. In this research, it was evident that a higher TTMP yield could be achieved using ST-treated cane molasses as a carbon source.

## 3. Materials and Methods

### 3.1. Bacterial Strain, Materials and Culture Medium

The bacterium was isolated from Daqu of Danquan distillery in Nandan county, Hechi city, Guangxi Autonomous Region. The strain can synthesize very large quantities of TTMP in high-temperature fermentation, which was named as *Bacillus* sp. TTMP20. Cane molasses, a by-product from sugar production, were obtained from a sugar factory in Guangxi Autonomous Region.

Growth medium consists of 10 g/L tryptone, 5 g/L NaCl, 5 g/L beef extract, pH = 7.2. The fermentation medium producing TTMP consists of (1) 170 mL/L treated molasses liquid, 10 g/L yeast powder, 30 g/L tryptone and 30 g/L (NH_4_)_2_HPO_4_. (2) 150 g treated molasses per L, 10 g/L yeast powder, 30 g/L tryptone and 30 g/L (NH_4_)_2_HPO_4_.

### 3.2. Pretreatment of Cane Molasses

Cane molasses, a by-product from sugar production, were obtained from a sugar factory in Guangxi Autonomous Region. Cane molasses was diluted with ultrapure water at a proportion of 1:1 (m:m), and then centrifuged at 8000 rpm for 10 min to remove sediment as a control. The following methods were selected to treat cane molasses. The diluted molasses with 1:1 (m:m) was used as raw material and calcium hydroxide was used to adjust the proper pH value.

(1) Sulfuric acid treatment process (ST). The diluted molasses was boiled for 30 min, cooled down quickly, and pH was adjusted to 1.0–2.0 with sulfuric acid. Then the mixture was left overnight. The pH of the supernatant after centrifugation was adjusted to 6.0 by calcium hydroxide. The mixture was kept at 60–70 °C for 30 min, and then centrifuged (8000 rpm, 10 min). The supernatant was used for fermentation after adding 0.8% active carbon to adsorb pigment.

(2) Phosphoric acid treatment process (PT). The pH of diluted molasses was adjusted to 1.0–2.0 by the addition of phosphoric acid, the other steps were same to that in sulfuric acid treatment process.

(3) Potassium ferrocyanide treatment process (PFT). Cane molasses was diluted with ultrapure water. Potassium ferrocyanide with final concentration of 0.1% (m:m) was added to the diluted molasses, controlling pH value of the solution to 6.5. Then heat up to 100 °C to condense for 30 min, add active carbon with 1% weight of reactant. Collect the supernatant for fermentation.

(4) Calcium phosphate tribasic treatment process (COT). Calcium phosphate tribasic was mixed with the diluted molasses according to the proportion of 8 g/100 mL at room temperature with stirring for 4 h on a magnetic stirrer. The supernatant was collected for fermentation.

(5) Combinational treatment process (CT). Boil the diluted molasses for 30 min, cool off the molasses quickly, and adjusted pH to 1.0–2.0 with sulfuric acid, then let the mixture stand overnight. After centrifugation, pH of the supernatant was adjusted to 5.5 by calcium hydroxide, then active carbon was added with 1% weight of reactant. At last, add the proper amount of potassium ferrocyanide with final concentration of 0.1% (m:m). Collected by centrifugation at 9000 g for 10 min. Calcium phosphate tribasic was mixed with the diluted molasses according to the proportion of 8 g/100 mL at room temperature with stirring for 4 h. Collect the supernatant for fermentation.

### 3.3. Analysis of Sugar Content and Acetoin Content

Polysaccharide content was determined by anthrone-sulfuric acid method. The content of reducing sugar was determined by DNS method [54]. Glucose content was determined by Glucose Content Assay Kit (Sangon Biotech, IL, USA) at 505 nm. Sucrose content was examined with Plant Sucrose Content Assay Kit (Sangon Biotech, IL, USA). Acetoin content was tested by Voges-Proskauer reaction [55].

### 3.4. Measurement of Cell Growth

Growth curve of the strain was determined by Microplate Reader at 600 nm.

### 3.5. HPLC Analysis

HPLC was used to analyze TTMP content. Agilent 5 TC-C18 (2) C18 Column (4.6 mm × 250 mm) was used for the separation. The fermentation broth of *Bacillus* sp. TTMP20 was centrifuged (12,000 rpm, 15 min), and the supernatant was diluted to an appropriate concentration, then filtered using 0.22 µm filter membrane. Mobile phase A (ultrapure water) was mixed with mobile phase B (methanol) according to the proportion (A:B = 30:70). HPLC was performed at a flow rate of 0.8 mL/min, and detected at 290 nm. The injection volume was 20 μL, and column temperature was maintained at 37 °C.

## 4. Conclusions

In this study, *Bacillus* sp. TTMP20 with high yield of TTMP was isolated from high-temperature Daqu of Danquan distillery. Five kinds of pretreatment methods (ST, PT, PFT, COT and CT) were used to treat cane molasses. Then treated molasses was used as carbon source for TTMP production by *Bacillus* sp. TTMP20.

In this study, different treatment methods of molasses were used. The substances added in different treatment methods were different, the process complexity was different, and the volume of the supernatant obtained after treatment was different. To better discuss the differences of molasses with different treatment methods in the production of TTMP, two methods were selected to design culture medium. The first preparation method was to take the same volume of treated molasses (170 mL/L) to prepare the medium for fermentation. In the medium prepared by this method, the molasses treated by PT method had the best fermentation effect, and the yield of TTMP was 208.8 mg/L. The second preparation method was to take six portions of 150 g molasses and obtain six portions of supernatant after the treatment of the six methods. Six portions of supernatant were used to prepare six portions of 1 L culture medium, then the strain was inoculated and fermented for 36 h. Using molasses (150 g) with ST pretreatment, the highest yield of TTMP was 895.13 mg/L, which was 183.18% higher than the control (316.1 mg/L). This study showed that molasses was a high-quality and cheap carbon source for TTMP production, which laid a foundation for industrial production of TTMP.

## Figures and Tables

**Table 1:** Chemical composition of molasses.

Composition	Content
Total sugars	37.77%
Reducing sugars	8.55%
pH	4.84
Ash	8.4%
Water	25.24%
Crude protein	13.25%

**Table 2:** Change in the composition of molasses after treatment.

Pretreatment Methods	Total Sugars (g/L)	Reducing Sugars (g/L)	Sucrose (g/L)	Glucose (g/L)	OD_600_
Diluted molasses (DM)	423.24 ± 57.11 ^a^	109.05 ± 19.45 ^bc^	45.22 ± 3.83 ^a^	32.8 ± 2.26 ^b^	1.56 ± 0.12 ^ab^
ST	401.54 ± 97.95 ^a^	142.57 ± 30.85 ^b^	38.59 ± 0.17 ^a^	34.32 ± 2.73 ^b^	2.08 ± 0.32 ^a^
PT	248.98 ± 16.61 ^b^	75.67 ± 8.23 ^c^	40.14 ± 4.62 ^a^	33.49 ± 0.31 ^b^	1.57 ± 0.84 ^ab^
PFT	345.63 ± 49.30 ^ab^	85.48 ± 10.39 ^c^	37.7 ± 3.21 ^a^	30.4 ± 0.80 ^b^	1.79 ± 0.10 ^a^
COT	456.67 ± 119.41 ^a^	113.53 ± 31.66 ^bc^	42.36 ± 5.77 ^a^	31.27 ± 5.88 ^b^	1.92 ± 0.05 ^a^
CT	312.53 ± 23.37 ^ab^	200 ± 17.22 ^a^	14.26 ± 1.09 ^b^	45.61 ± 0.87 ^a^	0.82 ± 0.21 ^b^

^a,b,c^ -Data with a same superscripted letter mean that there is no statistical difference in the same column.

## Data Availability

All data generated or analyzed during this study are included in this published article.

## Sample Availability

Samples of the compounds are available from the authors.

## Abbreviations

TTMP: 2:3,5,6-Tetramethylpyrazine; ST: Sulfuric acid treatment process; PT: Phosphoric acid treatment process; PFT: Potassium ferrocyanide treatment process; COT: Calcium phosphate tribasic treatment process; CT: Combinational treatment process
